# GDF-15 protects from macrophage accumulation in a mousemodel of advanced atherosclerosis

**DOI:** 10.1186/2047-783X-18-19

**Published:** 2013-06-24

**Authors:** Michael R Preusch, Matthias Baeuerle, Claudia Albrecht, Erwin Blessing, Marc Bischof, Hugo A Katus, Florian Bea

**Affiliations:** 1 Department of Internal Medicine, University of Heidelberg, Im Neuenheimer Feld 410, 69120 Heidelberg, Germany; 2 Department of Radiooncology, University of Heidelberg, Im Neuenheimer Feld 400, 69120 Heidelberg, Germany; 3 Freudenstadt Hospital, Karl-von-Hahn-Str. 120, 72 250 Freudenstadt, Germany

## Abstract

**Background:**

The cytokine growth differentiation factor-15 (GDF-15), a member of the TGF beta superfamily, has recently been discovered to play an important role in cardiovascular diseases. It is mostly expressed in macrophages of atherosclerotic lesions, but its impact on advanced atherosclerosis is still unknown. This study was performed to evaluate the effects of GDF-15 in an established mouse model of advanced atherosclerosis.

**Methods:**

Thirty-eight LDL receptor deficient mice received a lethal body radiation. Half of the group was transplanted with bone marrow of GDF-15 deficient mice. Nineteen mice were transplanted with bone marrow from wild-type controls. After 24 weeks on an atherogenic diet, animals were euthanized and sections of the aortic sinus were prepared. Lesion size and lesion composition, as well as macrophage content,were evaluated.

**Results:**

While demonstrating no difference in lesion size, LDL-receptor knockout mice transplanted with bone marrow from GDF-15 deficient mice showed enhanced macrophage accumulation and features of atherosclerotic plaque destabilization, such as thinning of fibrous caps. Immunostaining against intercellular adhesion molecule-1 further revealed an increased expression in mice receiving GDF-15-deficient bone marrow.

**Conclusions:**

This is the first study that demonstrates a protective role of GDF-15 in advanced atherosclerosis and macrophage accumulation, possibly due to the reduced expression of adhesion molecules.

## Background

Cytokines are known to play a key role in the development and progression of atherosclerosis [1]. Among these, members of the transforming growth factor-β (TGF-β) superfamily have been shown to contribute to the development of vascular inflammation [2,3]. Recently, growth differentiation factor-15 (GDF-15), a member of the TGF-β family, has been identified to participate in cardiovascular pathology. GDF-15, also known as macrophage inhibitory cytokine-1, prostate-derived factor or non-steroidal anti-inflammatory drug-activated gene-1, is a 12-kDa secreted protein (and a 25-kDa disulfide-linked dimer) which is, besides in placenta and prostate, not expressed under basal conditions. It can be induced by inflammation, injury, and malignancy [4,5]. Furthermore, GDF-15 is involved in apoptosis and cardiac hypertrophy, and can be induced by biomechanical stretch [6-9]. In addition, several clinical studies have investigated its role in cardiovascular diseases. GDF-15 is described as a novel biomarker with a high impact on risk stratification and prognostic value in myocardial infarction, chronic heart failure, and pulmonary embolism [10-13]. The mechanisms through which GDF-15 acts, however, seem complex and are still unclear. In atherosclerotic lesion development, most experimental studies have demonstrated the anti-atherogenic properties of TGF-β [14,15]; however, these have not been defined for the different members of the TGF-β superfamily [2,16]. Recently, a study by de Jager et al.demonstrated an anti-atherosclerotic effect of GDF-15 deficiency in low-density lipoprotein (LDL)r^−/−^ mice 4 and 12 weeks after initiation of a hyperlipidemic diet [17]. However, it is not known how GDF-15 acts in the advanced stages of atherosclerosis that we often find in human disease. In the present study, we tested whether GDF-15 alters lesion size and lesion composition in an advanced stage of atherosclerosis.

## Methods

### Animals and bone marrow transplantation

Eight-week-old female LDL-receptor^−/−^ mice (LDLr^−/−^, background C57/BL/6; Jackson Laboratory, Bar Harbor, USA; n=38) received lethal body irradiation at a dose of 9 Gy. Half of the group (n=19) was transplanted with bone marrow of mice (n=5) with a GDF-15 knock-out [18]. Nineteen LDLr^−/−^ mice were used as controls, which were transplanted with bone marrow of wild-type mice (C57/BL/6CR). After transplantation, mice were fed a high fat western-type diet (Altromin, Lage/Germany; Nr. 11320010: 0.15% cholesterol) for 24 weeks. Animals were kept within the animal care facility of the University of Heidelberg. The investigation conforms to the *Guide for the Care and Use of Laboratory Animals* published by the US National Institutes of Health (NIH Publication No. 85–23, revised 1996). The housing and care and procedures in the study were performed in accordance with the guidelines and regulations composed by the Animal Care Committee of the University of Heidelberg and approved by the Regierungspraesidium Karlsruhe.

### Animal sacrifice and preparation of tissues

After 24 weeks on a high cholesterol Western-type diet, mice were heavily sedated (Avertin, Aldrich, Milwaukee, USA), blood was collected from the inferior vena cava, and the animals were sacrificed by exsanguination (fasted 3 h prior to sacrifice). The animals were perfused with 10 mL phosphate-buffered saline, followed by a perfusion with 4% buffered formalin via the left ventricle. The entire heart from each animal was dissected out, embedded in paraffin, and the aortic sinus was serially sectioned (5 μm). Every fifth section was stained with a modified Movat’s pentachrome stain [19].

### Assessment of chimerism

The reconstitution of the transplanted bone marrow was determined by PCR on liver and spleen tissue.

### Determination of plasma lipid concentration

Total serum cholesterol, high-density lipoprotein (HDL), LDL cholesterol, and triglycerides were determined enzymatically in heparinized plasma.

### Evaluation of lesion size and lesion composition

Two investigators who were blinded to the study protocol determined the cross-sectional area of the lesion in each section by using computer-assisted morphometry (Image Pro, Media Cybernetics, Silver Spring, USA); this is reported as mean plaque area per animal (data expressed in μm^2^). We further evaluated each section for characteristic features of plaque morphology/composition: thickness of the fibrous cap (presented as μm), size of the necrotic core (a large necrotic core was defined as occupying more than 50% of the plaque’s volume and was measured by computer). Calcification was determined using von Kossa staining [20].

### Immunohistochemistry

Detection of monocytes/macrophages was performed using monoclonal goat anti-mouse antibody (anti-Mac-2, Accurate, NY, USA) and detection of ICAM-1 by using a polyclonal antibody (Santa Cruz Biotech, CA, USA). Sections were incubated with the biotinylated secondary antibody, rinsed three times with PBS, and incubated for 10 minutes with streptavidin at room temperature. AEC-chromogen substrate (Invitrogen, Karlsruhe, Germany) was used for visualization. The extent of positive staining within the lesions was determined using computer-assisted morphometry and is presented as ratio stained area/total lesion area (Image Pro, Media Cybernetics, Silver Spring, USA).

### Statistical analysis

All data were expressed as mean ± SEM. Differences between means in plasma lipid profiles were determined with the two-tailed unpaired student’s *t*-test. For analysis of plaque morphometry and areas of positive staining, groups were compared using the two-tailed Mann–Whitney U test. For evaluation of plaque morphology, groups were compared using the χ^2^test. A *p* value <0.05 was considered statistically significant.

## Results

### Effect of bone marrow transplantation

Polymerase chain reaction analysis of the bone marrow demonstrated a complete conversion of the original LDLr^−/−^ genotype to the donors’ type, indicating that the bone marrow population had been reconstituted (data not shown). There were no differences in body weight and mortality between the groups.

### Effect on plasma lipid level and body weight

There were no significant differences in total cholesterol, LDL, HDL, and triglycerides between mice that received GDF-15^−/−^ bone marrow and controls. Furthermore, there was no difference in body weight (Table 1).

**Table 1:** **Distribution of body weight, total serum cholesterol, LDL cholesterol, HDL cholesterol, and serum triglycerides of recipients of GDF-15**^**−/−**^**bone marrow (GDF-15**^**−/−**^**) and wild-type controls (GDF-15**^**+/+**^**) (*p *was non-significant)**

	**Body weight (g)**	**Total cholesterol (mg/dL)**	**LDL cholesterol (mg/dL)**	**HDL cholesterol (mg/dL)**	**Triglycerides (mg/dL)**	**Lesion size (μm**^**2**^**)**
**GDF-15 **^**+/+ **^**(WT)(n=15)**	28±1	351±28	293±26	25±2	159±20	246,566±14,788^†^
**GDF-15 **^**−/− **^**(KO)(n=17)**	29±1	364±15	309±14	28±3	134±5	203,079±17,898^†^

Quantitative evaluation of lesion size within the aortic root also showed no significant difference between the groups (*p*=0.08); Mean±SEM; ^**†**^*p*=0.08.

WT: wild-type (recipients of bone marrow from C57/Black 6 control mice); KO: knock-out (recipients of bone marrow from GDF-15 negative mice).

### Mean lesion area

After 24 weeks on the western type diet, the extent of atherosclerotic lesion development in the aortic sinus was evaluated. We could not detect any significant difference in lesion size (GDF-15^+/+^246.566±14.788 μm^2^*versus* GDF-15^−/−^203.079±17.898 μm^2^, *p*= 0.08; Table 1).

### Enhanced macrophage content in GDF-15 deficient mice

**Figure 1:**
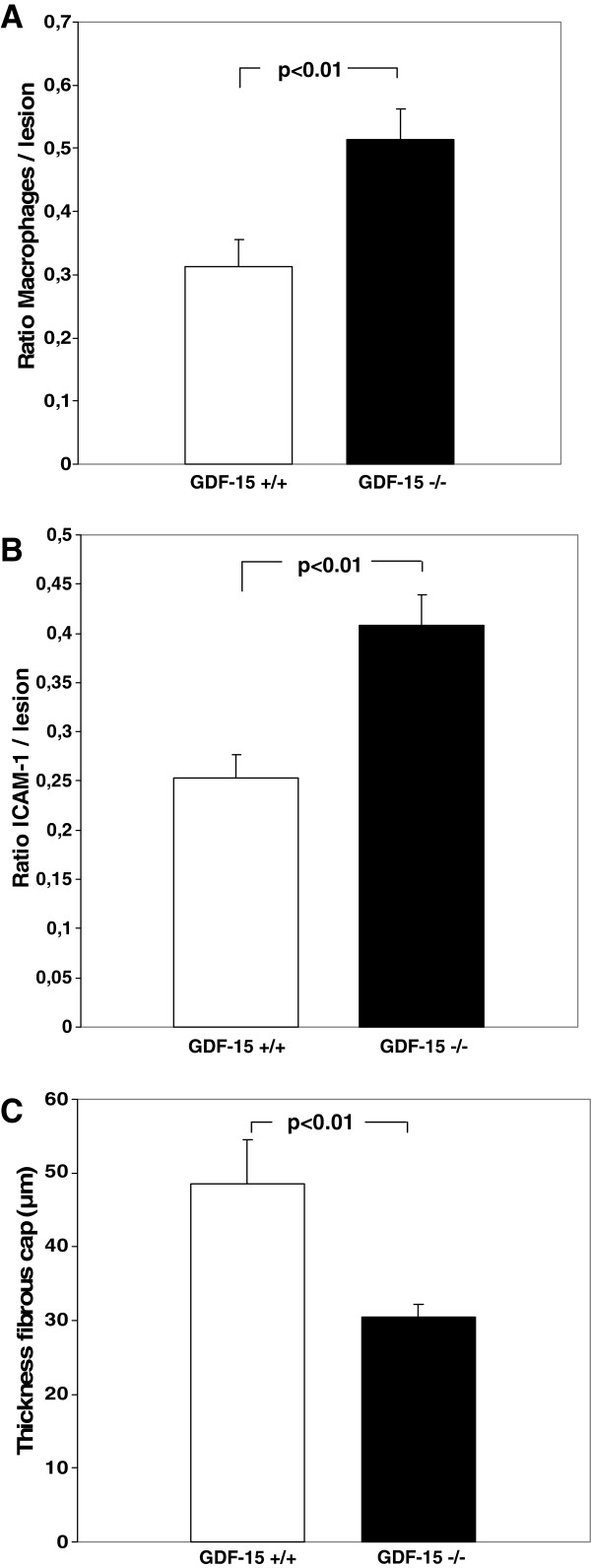
**Enhanced macrophage accumulation and ICAM-1 expression within the atherosclerotic lesions of mice transplanted with bone marrow from GDF-15**^**−/− **^**donors. (A)** Video-microscopic evaluation of positive staining for macrophages (Mac-2-staining) showed significant enhanced macrophage accumulation within the atherosclerotic lesions of LDLr^−/−^ mice transplanted with bone marrow from GDF-15^−/−^ mice in comparison to wild-type controls (*p*<0.01). Data are presented as ratio positive macrophage staining/lesion; Mean±SEM. **(B)** Video-microscopic evaluation of positive staining for ICAM-1 demonstrated a significant enhanced expression within atherosclerotic lesions of LDLr^−/−^ mice transplanted with bone marrow from GDF-15^−/−^ mice compared to wild-type controls (*p*<0.01). Data are presented as ratio positive ICAM-1 staining/lesion; Mean±SEM. **(C)** Atherosclerotic lesions of LDL^−/−^ mice transplanted with bone marrow from GDF-15^−/−^ donors (*p*<0.01) showed a significantly thinner fibrous cap than controls. Data are presented as μm thickness; Mean±SEM.

**Figure 2:**
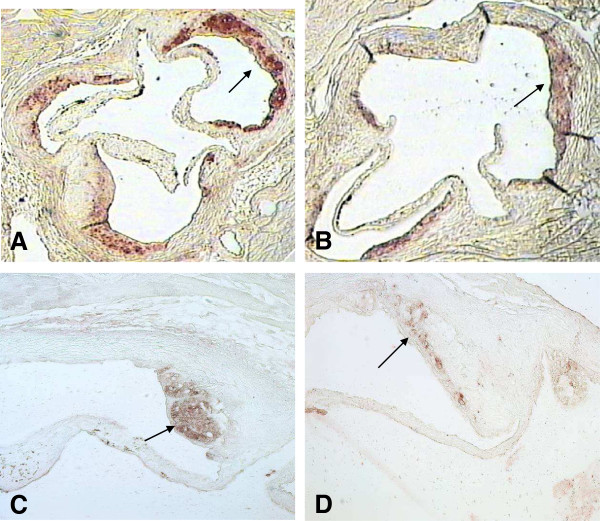
**Macrophage staining of the aortic root of a LDLr**^**−/−**^**mouse, transplanted with bone marrow from a GDF-15**^**−/−**^**donor (A) demonstrated an enhanced macrophage accumulation (arrows) within the atherosclerotic lesion compared to a control animal (B).** Staining for ICAM-1 furthermore showed an increased expression (arrows) in animals transplanted with GDF-15 deficient bone marrow **(C)** in contrast to controls **(D)**. Pictures demonstrate a colocalization of macrophages and ICAM-1.

After 24 weeks on the western-type diet, we were able to demonstrate macrophage rich lesions and enhanced foam cell formation evaluated by macrophage staining in both groups. Mice transplanted with bone marrow of GDF-15^−/−^ donors showed enhanced macrophage accumulation within atherosclerotic lesions (0.51 *versus* 0.31;*p*<0.01; Figures 1A and 2A).

### Mice transplanted with GDF-15 deficient bone marrow showed enhanced expression of intercellular adhesion molecule-1 (ICAM-1)

ICAM-1 staining was enhanced in atherosclerotic lesions of chimeric mice (0.41 *versus* 0.25 in wild-type controls, *p*<0.01; Figures 1B and 2B).

### Features of lesion composition

Video-microscopic evaluation of features of lesion-destabilization showed significantly more thinning of the fibrous cap in LDLr^−/−^ mice transplanted with bone marrow from GDF-15^−/−^ mice than in controls (48.5 μm *versus* 30.5 μm, *p*<0.01; Figure 1C). We could not detect any difference in size of the necrotic core or calcification within the lesions (data not shown).

## Discussion

Recent studies have hypothesized a crucial role of the cytokine GDF-15 in cardiovascular diseases. While clinical investigations demonstrate GDF-15 as a parameter for risk stratification in myocardial infarction and heart failure, experimental studies show a cardio-protective effect in ischemia and reperfusion [7,10-13];furthermore, GDF-15 is correlated with systemic inflammation [21]. These data suggest an involvement of GDF-15 in the initiation and progression of atherosclerosis. Recently, de Jager et al. demonstrated an anti-atherosclerotic effect of GDF-15 deficiency in a mouse model of atherosclerosis [17]. The authors used LDLr^−/−^ mice transplanted with GDF-15-deficient bone marrow. In this study, GDF-15 deficiency resulted in a reduction of early atherosclerotic lesion size after 4 weeks on a high cholesterol western-type diet. After 12 weeks, no differences in lesion size could be detected. Using mice following 24 weeks on a western-type diet, we focused on more advanced and complex lesions to model late-stage disease. It is known that lesions in mice become quite complex with increased duration of feeding [22]. We could not detect any differences in lesion size, but in contrast to the findings of de Jager et al., our data demonstrated a pro-inflammatory plaque phenotype in mice transplanted with bone marrow from GDF-15^−/−^ donors with enhanced macrophage accumulation [17]. In the present study macrophages were identified by using a Mac-2 antibody, which is an appropriate staining used in many LDLr^−/−^ mouse studies. We cannot exclude that staining for other macrophage markers will identify different subpopulations of macrophages with different results. The increase seen in our study was accompanied by enhanced expression of ICAM-1 within lesions.

Monocyte/macrophage recruitment is dependent on adhesion molecules [23]. ICAM-1 is mostly expressed by endothelial cells but also in macrophages within atherosclerotic lesions and it is supposed to be involved in foam cell transformation of monocytes and therefore contributes to changes in lesion vulnerability [24,25].

Our data also confirm a correlation between enhanced macrophage content and signs of the vulnerable plaque determined by the thickness of fibrous caps. This is in line with autopsy findings of ruptured plaques in human [26]. Macrophages excrete an excess of matrix-degrading enzymes and macrophage-rich lesions, and therefore most likely undergo thinning of the fibrous caps and subsequent enhanced vulnerability followed by plaque rupture [27,28]. However, our findings of an association between GDF-15 deficiency and reduced plaque stability are in contrast to the findings of de Jager et al., where a decreased necrotic core formation in GDF-15^−/−^ chimera is reported [17]. It is known that at one point in atherosclerotic lesion development, changes in plaque composition but not progression of size,are dominating. The increase in macrophages and the subsequent increase in inner-plaque inflammation finally results in a reduction of plaque stability. Other than the effect on fibrous caps, we could not detect any differences in other features of lesion destabilization, which might also be due to the duration of the study and the animal model since it is known that differences in necrotic core and hemorrhage are more common in brachiocephalic arteries in apoE^−/−^ mice [29].

There are several limitations to our study. Investigating atherosclerotic lesions in LDLr^−/−^ mice is mostly done in the aortic root, which is not a typical lesion location. It is known as a model of early stages in atherosclerosis and does not show much progress in late-stage disease [30,31]. We did not focus on the onset of atherosclerotic changes within the vascular wall such as lipid accumulation in younger mice. Evaluation of fibrous caps was performed morphometrically as in many LDLr^−/−^ mouse studies. Given the amount of tissue obtained, we were not able to stain for other parameters such as the differences in collagen content. Further, we do not know if bone-marrow transplantation has an effect on other cytokines, the immunosystem, or metabolism, which is an important factor in atherosclerosis. Recently, it has been shown that GDF-15 is a key regulator in anorexia, and weight and fat loss [32]. However, lipid levels and body weight in our study were equally distributed. We could not detect any further change in lethality after transplantation.

## Conclusions

In conclusion, this is the first study evaluating the effects of GDF-15 in advanced stages of atherosclerosis. We were able to demonstrate a GDF-15-dependent inhibition of macrophage adhesion and accumulation in an atherosclerotic LDLr^−/−^ mouse model. This effect may contribute to changes in lesion vulnerability such as thinning of fibrous caps and potential plaque rupture.

## Abbreviations

GDF-15: Growth differentiation factor-15; HDL: High-density lipoprotein; LDL: Low-density lipoprotein; TGF-β: Transforming growth factor-β.

## Competing interest

All authors declare that they have no competing interests.

## Authors’ contribution

MB and CA carried out the immunostaining, PCR and lesion analyses. MBi performed the body irradiation. MRP, MB, EB, and FB, participated in the design of the study and performed the statistical analysis. MP, HAK, and FB conceived the study and participated in its design and coordination, and helped to draft the manuscript. All authors read and approved the final manuscript.
